# Explainable transfer learning ensemble AI model for lung ultrasound pneumothorax detection with expert benchmark

**DOI:** 10.1186/s13049-026-01614-4

**Published:** 2026-04-17

**Authors:** Gábor Orosz, Róbert Zsolt Szabó, Marcell Szabó, Pál Gyombolai, József T. Tóth, Tamás Ruttkay, Tamás Ferenci, Tamás Ungi, Gábor Fichtinger, Tamás Haidegger

**Affiliations:** 1 Department of Military, Disaster and Law Enforcement Medicine, Semmelweis University, P.O.B. 2, Budapest, 1428 Hungary; 2 Department of Anesthesiology and Intensive Therapy, Semmelweis University, P.O.B. 2, Budapest, H-1428 Hungary; 3 University Research and Innovation Center, John von Neumann Faculty of Informatics, Óbuda University, Bécsi Út 96/B, Budapest, Hungary; 4 Department of Surgery, Transplantation and Gastroenterology, Semmelweis University, P.O.B. 2, Budapest, 1428 Hungary; 5 Department of Anaesthesiology, Intensive Care and Pain Medicine, University Hospital Limerick, St Nessan’s Road, Dooradoyle, Limerick, V94 F858 Ireland; 6 Department of Intensive Therapy, Semmelweis University, P.O.B. 2, Budapest, 1428 Hungary; 7 PSI Pain Clinic, MEDCITY Health Center, Budapest, Hungary; 8 Department of Anatomy, Histology and Embryology, Semmelweis University, P.O.B. 2, Budapest, 1428 Hungary; 9 Physiological Controls Research Center, Óbuda University, Bécsi Út 96/B, Budapest, Hungary; 10 Department of Statistics, Corvinus University of Budapest, Fővám Tér 8, Budapest, Hungary; 11 Laboratory for Percutaneous Surgery, School of Computing, Queen’s University, Kingston, ON 2K7L 2N8 Canada; 12 Austrian Center for Medical Innovation and Technology, Viktor-Kalpan-Str. 2., Wiener Neustadt, 2700 Austria

**Keywords:** Lung ultrasound, Pneumothorax detection, Explainable artificial intelligence, Transfer learning, Ensemble learning, M-mode ultrasound imaging, Expert benchmarking, Deployable AI tool, Critical care

## Abstract

**Background:**

Lung ultrasound is essential for rapid, radiation-free bedside pneumothorax diagnosis but limited by variability in human interpretation. Key gaps include insufficiently large and diverse human datasets, inconsistent image acquisition, lack of rigorous expert benchmarking, and inadequate clinical interpretability of existing artificial intelligence models. We aimed to develop and validate a robust, explainable artificial intelligence (AI) ensemble model addressing these critical gaps.

**Methods:**

With our multidisciplinary team, we developed an explainable soft-voting ensemble model trained on 1,856 diverse ultrasound clips from critically ill patients, healthy volunteers, and tailored cadaver models. Model interpretability was ensured using visualization, with heatmaps validated by expert clinicians. The model’s diagnostic performance was rigorously benchmarked against 11 experienced clinicians using an independent, balanced test set. Statistical analyses included sensitivity, specificity and inter-rater reliability.

**Results:**

The ensemble model achieved 100% sensitivity (95% CI: 85·8%-100·0%) and 100% specificity (95% CI: 85·8%-100·0%), surpassing expert sensitivity and specificity. Diagnostic performance of experts significantly differed by ultrasound mode, with notably lower specificity in M-mode imaging (*p* < 0·001). The AI consistently maintained perfect sensitivity and significantly reduced false positives compared to clinicians across all conditions, including challenging diagnostic scenarios (e.g., subtle pleural motions), and showed excellent generalizability to both cadaveric and clinical cases.

**Conclusions:**

Our explainable AI ensemble robustly matches the consensus-level performance of an expert "committee," significantly reducing diagnostic variability and false-positive diagnoses. This AI tool can serve as a critical second reader, standardize clinical decisions at the bedside, and substantially improve patient safety.

**Supplementary Information:**

The online version contains supplementary material available at 10.1186/s13049-026-01614-4.

## Highlights

- AI ensemble achieved 100% sensitivity and specificity on lung ultrasound data
- Statistically outperformed 11 experts in specificity across imaging modes
- Trained on diverse ICU, cadaver, and healthy volunteer ultrasound datasets
- Explainable Grad-CAM++ visualizations matched expert clinical interpretation
- Real-time AI deployment demonstrated for bedside pneumothorax detection

**Supplementary Information:**

The online version contains supplementary material available at 10.1186/s13049-026-01614-4.

## Background

Pneumothorax (PTX) in mechanically ventilated patients can escalate to life-threatening tension PTX within minutes, necessitating immediate diagnosis and intervention [1]. PTX causes impaired venous return and can lead to cardiovascular collapse if not promptly relieved by needle decompression or chest tube placement [2]. Traditional signs and bedside examinations are often unreliable in critically ill patients [3], and chest X-rays (especially portable AP views in ICU) have suboptimal sensitivity [4, 5]. Thus, there is a pressing clinical need for fast and accurate bedside diagnostic tools in the intensive care setting.

**Fig. 1:**
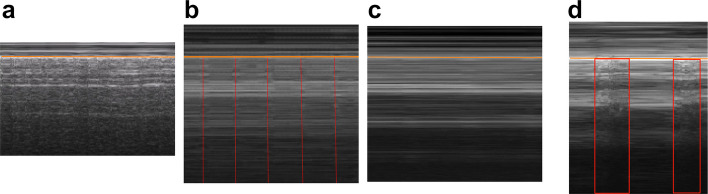
Representative M-mode lung ultrasound patterns demonstrating the four relevant classes of pleural motion (a to d from left to right). Horizontal “orange” line: pleural line. **a** Lung sliding (seashore sign) – normal lung motion pattern, ruling out PTX. **b** Lung pulse (T-lines sign) – indicates cardiac-induced pleural motion without respiratory sliding, also ruling out PTX. See vertical “red” lines originating from the pleural line. **c** Absence of lung sliding (barcode sign) – indicates lack of pleural motion, consistent with PTX. **d** Lung point – clear boundary between moving and non-moving pleural segments, pathognomonic for PTX. Vertical “red” boxes: normal motion with clear border with non-moving segments

Lung ultrasound (LUS) has emerged as an ideal modality for PTX detection in acute care, being non-invasive, radiation-free [6], portable, and yielding immediate results with diagnostic precision approaching that of CT scans [7]. Conventionally, B-mode ultrasound (two-dimensional brightness mode) is used following standardized protocols to identify sonographic signs of PTX (e.g. absence of lung sliding, presence of non-A artifacts) [8, 9]. M-mode ultrasound (motion mode) can complement B-mode by capturing movement of structures over time as a static trace; for instance, a normal lung sliding produces a “seashore sign” on M-mode, whereas PTX yields a “barcode sign”, subtle movements appear as “T-lines” indicating lung pulse [10] (Fig. 1: Representative M-mode lung ultrasound patterns demonstrating the four relevant classes of pleural motion) [8]. Emerging evidence suggests M-mode may be especially useful in certain scenarios (e.g. patients with subcutaneous emphysema obscuring B-mode) [11, 12]. However, direct comparisons of B-mode vs. M-mode effectiveness in PTX diagnosis remain underrepresented in the literature.

Artificial intelligence has increasingly been applied to medical imaging, including LUS for PTX [13, 14]. Prior studies have explored machine learning models on ultrasound data [15–17], including using M-mode images derived from B-mode videos [18, 19]. Notably, many earlier works relied on simulations or animal models, which may not capture the full complexity of human PTX presentations [20].

Key gaps in current research include the limited size and diversity of human datasets, inconsistent image acquisition protocols, and a lack of validation against expert clinician performance. There is also a tendency to focus solely on algorithm metrics without assessing clinical concordance [21].

To address these gaps, we developed a robust AI-based diagnostic system for PTX in LUS and rigorously evaluated it against experienced human clinicians. Our approach leverages a soft-voting ensemble [22, 23] of five CNN architectures (ResNet [24], Inception v3 [25], Xception [26], Inception-ResNet-v2 [27], and VGG16 [28]) with transfer learning [29], fine-tuned on a large real-world LUS dataset. Importantly, we incorporate both B-mode and derived M-mode images into the analysis. We also convened an expert panel (11 LUS experts with > ten years of experience each) to create a human performance benchmark.

According to our best knowledge this study is the first to combine such a diverse clinical LUS dataset (including living patient and cadaver scans, normal and pathological findings) with an advanced ensemble AI model and direct comparison to multiple human experts.

Our hypothesis was that the ensemble AI model could achieve diagnostic performance on par with or exceeding that of seasoned clinicians, thereby demonstrating the feasibility of AI-assisted PTX diagnosis in critical care. Additionally, by using Grad-CAM + + (Gradient-weighted Class Activation Mapping) for explainability, we aimed to ensure the model’s decisions are interpretable and clinically trustworthy.

## Materials and methods

**Fig. 2:**
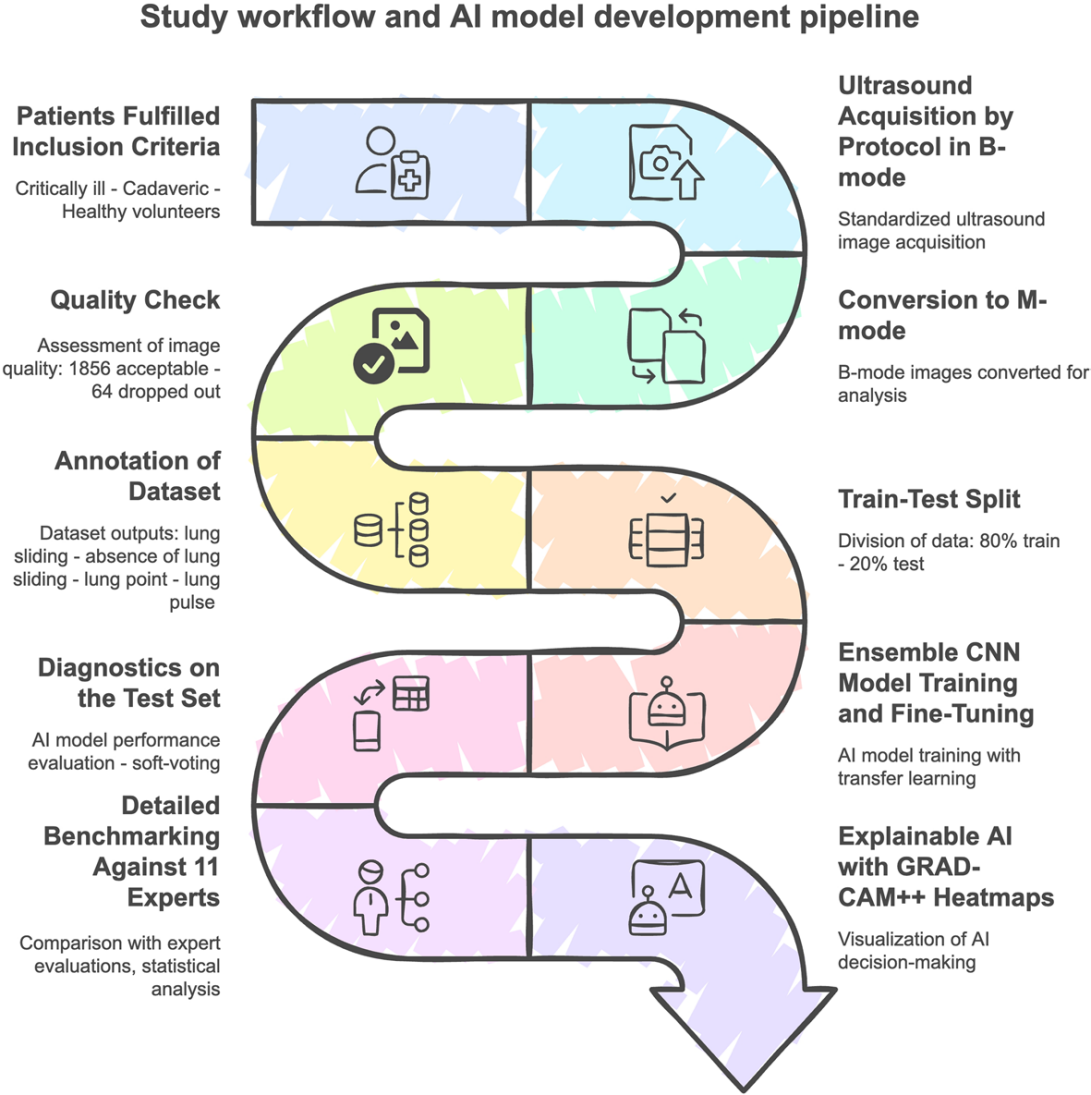
Study workflow and AI model development pipeline. The figure illustrates the step-by-step methodological approach applied throughout the study, including patient selection (critically ill patients, tailored cadaveric models, and healthy volunteers), standardized lung ultrasound data acquisition in B-mode, subsequent conversion to M-mode, rigorous quality assessment, and dataset annotation. Following an 80%-20% train-test data split, an ensemble of convolutional neural networks (CNNs) employing transfer learning was trained and fi ne-tuned. The model's diagnostic accuracy was then evaluated using the independent test set and comprehensively benchmarked against an expert panel of clinicians. Diagnostic interpretability was enhanced by implementing Gradient-weighted Class Activation Mapping (Grad-CAM++), facilitating visualization of image regions critical for AI-driven decisions

The complete workflow of the study, including data acquisition, processing, ensemble CNN model development with transfer learning, and detailed benchmarking against human experts, is comprehensively illustrated in Fig. 2: Study workflow and AI model development pipeline.

### Data collection and ethics

A comprehensive LUS dataset was prospectively collected with institutional ethics approval (*No. 303/2021*) and in compliance with GDPR. The data originated from a university hospital ICU during and after the COVID-19 pandemic, providing a wide variety of respiratory pathologies. This includes cases of acute respiratory failure due to COVID-19 and other causes, ensuring diversity in lung conditions. In addition to ICU patients, human cadaveric specimens (donated to Semmelweis University’s Human Anatomy Department for research) were used to obtain definitive PTX images under controlled conditions, and healthy volunteers (physicians) contributed normal LUS recordings (with variations like breath-holding to simulate certain signs). The cadaveric pneumothorax cases were prepared using a specialized protocol developed by our team (previously published by our group) [30]. As a result, it produces highly realistic sonographic manifestations of PTX and yields high-quality PTX ultrasound images.

All subjects were adults (> 18 years), and patient identifiers were removed (pseudo-anonymized) to protect privacy. Bedside lung ultrasound assessment is a declared part of the daily care at the study site [31, 32], constructed by the authors of this study and therefore no additional consent was needed. The cadavers included in the study were donated specifically for educational and research purposes to the Department of Anatomy, Histology and Embryology at Semmelweis University. Additional ethical review and approval was not required for the use of donated human cadaveric tissue in accordance with the local legislation and institutional requirements. All healthy volunteer provided informed consent for ultrasound examination, anonymized data collection, storage, and subsequent analysis and use for research purposes.

#### Dataset accounting and cadaver grouping

The final dataset comprised 1,856 M-mode images from 114 unique subjects. PTX-positive material included 23 cadavers scanned at 12 standardized thoracic points with three controlled inflations of 200, 400, and 600 mL air per subjects (828 pre-drop-out clips) and 2 live patients with confirmed PTX (12 measurements total; + 12 clips). PTX-negative material comprised 79 living patients (946 pre-drop-out seashore clips) and 10 healthy volunteers (120 pre-drop-out lung-pulse clips). After technical exclusions (14 PTX-positive, 28 seashore, 8 lung-pulse), 826 PTX-positive, 918 PTX-negative seashore, and 112 lung-pulse clips remained (1,856 total). All data partitions were performed at the subject level: all clips/images from a given subject (patient, volunteer, or cadaver) were assigned to a single split. For cadavers, the 200/400/600 mL acquisitions were treated as three readings of the same instance, not separate subjects. Accordingly, all three inflation-level acquisitions (200/400/600 mL) from a given cadaver were always kept within the same dataset split (training or hold-out test), preventing any cross-split leakage.

### Ultrasound imaging protocol

LUS videos and images were acquired using a standardized protocol developed by our team [31]—in line with the latest international guidelines, ensuring consistency across all scans [33, 34].

Each ICU patient dataset included ultrasound clips from multiple lung regions, later confirmed by reference standards (CT scans or expert review) for presence or absence of PTX. For clinical PTX-positive cases, lung ultrasound data were collected immediately after diagnosis was confirmed by chest CT scanning and prior to clinical intervention. For cadaveric cases, pneumothorax was artificially induced using our previously described protocol. Once the artificial PTX was created, ultrasound was first utilized to precisely identify the pleural borders and the exact extent of the PTX. These borders were marked externally on the cadaver’s skin with permanent ink. Subsequently, small metallic skin pins were placed directly over these markings to facilitate precise identification of PTX borders during CT scanning. Given the unique access to an on-site CT scanner at the Human Anatomy Department, cadavers underwent immediate CT scanning to verify that the marked borders accurately represented the PTX area (FIDEX GT, Animage LLC). An independent radiologist, blinded to the study's objectives, reviewed and confirmed the CT scans for the presence and correct delineation of PTX relative to the metallic markers. Following CT verification, ultrasound images representing confirmed PTX regions were systematically collected. The cadavers were mechanically ventilated using standardized settings (volume-controlled ventilation, tidal volume of 5 mL/kg body weight, and PEEP of 5 cmH₂O). All additional ultrasound data used in this study had been previously collected and categorized according to consensus from an expert panel, as detailed in our earlier published studies [35].

Key sonographic findings were categorized into four diagnostic classes relevant to PTX assessment, in line with international guidelines [33, 34, 36] (see Fig. 1):

- Lung sliding: Normal pleural movement indicating no PTX. This class included various underlying pathologies (e.g. pneumonia, adult respiratory distress syndrome, effusions, chronic obstructive pulmonary disease) but no PTX, confirmed by CT scan or chest X-ray and expert review. In M-mode, normal lung sliding corresponds to the classic “seashore sign”.
- Barcode sign and Lung point: The lung point is considered pathognomonic for pneumothorax. Barcode sign (stripes with no motion) indicates absent lung sliding and is strongly suggestive of PTX but is not definitive on its own, as it may also occur with, for example, pleurodesis, or pleural adhesions. Clips and images showing the barcode sign or lung point (transition point between sliding and no sliding) were conﬁrmed with concurrent CT scans, providing high-ﬁdelity PTX examples.
- Lung pulse: Subtle rhythmic pleural movement from cardiac oscillations, seen when lung sliding is absent but the lung is still in contact (e.g. mainstem intubation, apnea or breath-holding). We included lung pulse cases from breath-holding volunteers to ensure the model learns to distinguish lung pulse from true PTX.

Various nuanced LUS patterns (e.g. “minimal” sliding, Avicenna sign, pseudo A’-profile, and other artifacts) [8] were also represented to make the dataset reflective of real-world complexity. In total, the curated dataset comprised 1,856 annotated ultrasound instances. Each instance had an associated label (one of the four classes above). The dataset was randomly split into a training set (1,485 images, 80%) and a hold-out test set (371 images, 20%) for final evaluation.

### M-mode image generation

We generated M-mode images post hoc from B-mode videos by extracting a fixed vertical line of pixel intensities across frames and concatenating these over time—an established approach that reproduces machine-generated M-mode traces [18, 37]. In our pipeline, this standardized the input to a 256 × 256 single-image representation for classification and ensured one M-mode image per clip. To minimize dependency and leakage, multiple M-modes per clip were not created, and all data partitions were at the subject level, with cadaveric model subjects grouped across their 200/400/600 mL acquisitions into a single instance. We utilized this approach because, with this slight modification of the ultrasound imaging concept, we effectively transformed a complex bioengineering problem of interpreting dynamic pleural motion into a simplified, manageable two-dimensional image classification task, optimizing diagnostic accuracy for the AI model.

### AI model architecture

We developed a soft-voting ensemble of five CNN models, choosing architectures that performed best in our preliminary experiments: ResNet, Inception v3, Xception, Inception-ResNet-v2, and VGG16. Each model was initialized with ImageNet pre-trained weights to leverage learned visual features (transfer learning) [38, 39].

We employed fine-tuning: after replacing the final classification layer of each network to output our four classes, we trained not only this new layer but also continued to adjust the weights of the pre-trained layers (as opposed to freezing them). This strategy allows adaptation of low-level and mid-level features to the ultrasound domain, and was found to slightly outperform feature-extraction-only in initial tests. All networks were trained on the training set with data augmentation and optimized using the Adam optimizer. We used five-fold cross-validation on the training set to tune hyperparameters (batch size 32, learning rate 10^–4^, 30 epochs per fold), using categorical cross-entropy loss. After cross-validation, each model was re-trained on the entire training set (with the chosen hyperparameters) and then used for ensemble prediction on the test set.

In the ensemble’s soft-voting scheme, each model produces a probability distribution across the four classes for a given input. These probability outputs are averaged, and the class with the highest average probability is taken as the ensemble’s prediction. Soft voting effectively weights each model’s contribution by its confidence, and can yield a final decision that isn’t just a simple majority vote (unlike “hard” voting) [40]. This method was chosen for its improved reliability in our context, as it can capture consensus among models even if they disagree in discrete class votes. By combining multiple architectures, the ensemble aims to mitigate individual model biases and reduce overfitting, capitalizing on the “wisdom of the committee” to improve overall robustness.

### Explainability with Grad-CAM + + 

**Fig. 3:**
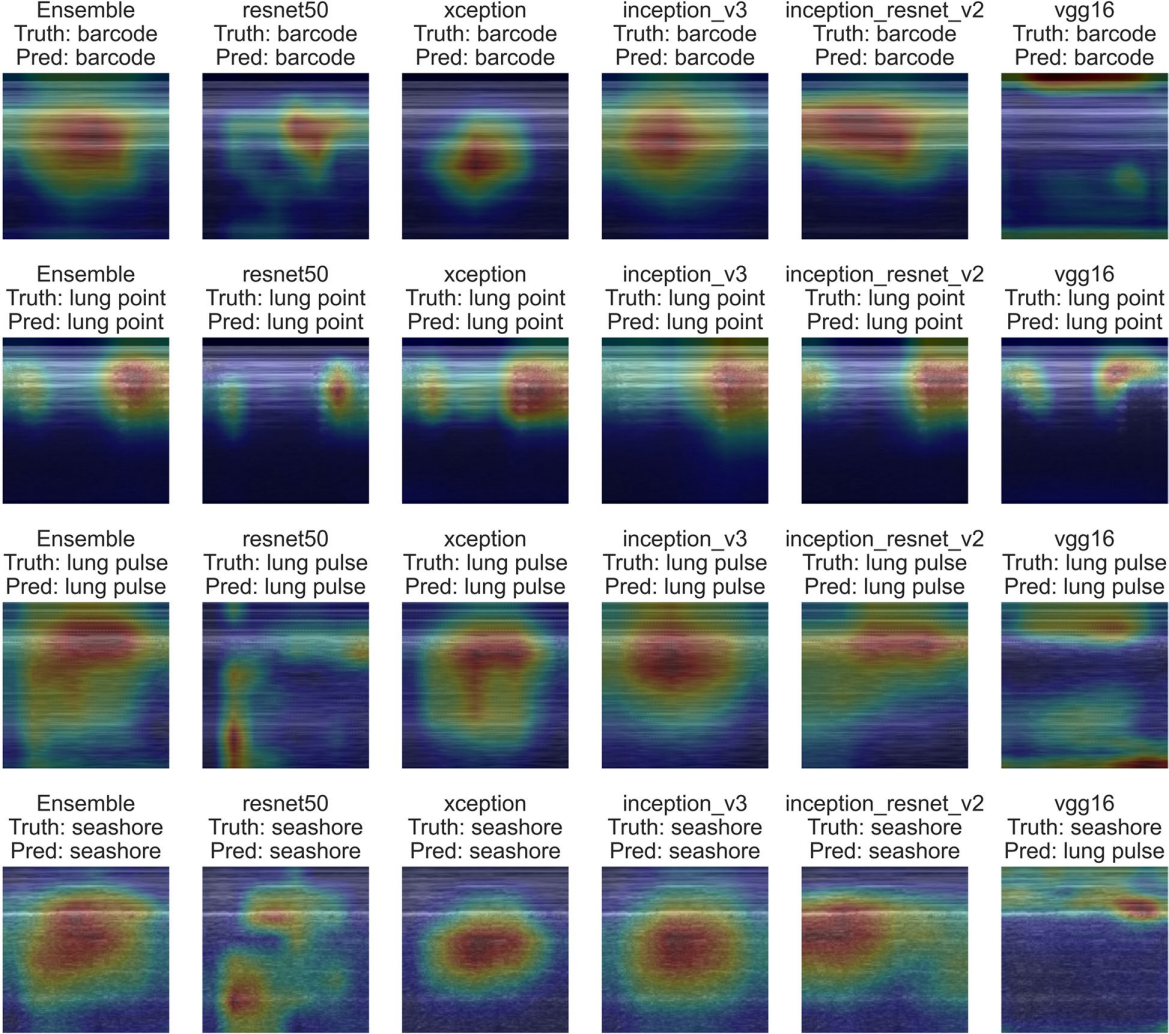
Grad-CAM++ heatmaps illustrating explainable AI predictions for pleural motion classification. Gradient-weighted Class Activation Mapping (Grad-CAM++) heatmaps visualizing areas of highest influence on model predictions for representative M-mode lung ultrasound images. Four distinct classes of pleural motion are shown (from top to bottom): absence of lung sliding (barcode sign), lung point, lung pulse (T-lines sign), and lung sliding (seashore sign). The heatmaps demonstrate predictions from each of the individual convolutional neural network models (ResNet50, Xception, Inception_v3, Inception_ResNet_v2, and VGG16), as well as the combined ensemble model (leftmost column). Red and yellow regions indicate stronger contributions to the classification decisions. Grad-CAM++ was chosen for its robust handling of neuron saturation and precise feature localization, essential for interpreting medical imaging predictions. Heatmaps were generated using the MONAI library's implementation of Grad-CAM++

To ensure the AI’s decisions are interpretable, we incorporated Gradient-weighted Class Activation Mapping (Grad-CAM + +) for post-hoc explanation of predictions. For each input M-mode image, Grad-CAM + + highlights the image regions that most strongly influenced the model’s classification. By averaging activation maps from all five models, we also produced an ensemble attention map indicating overall areas of importance. We chose the Grad- CAM + + variant [41] for its improved handling of neuron saturation and clearer localization of features. This is particularly important in medical imaging, where understanding why the AI labeled an image as PTX (e.g., highlighting the absent lung sliding under the pleural line region) is crucial for clinician trust and adoption. The MONAI library’s implementation [42] of Grad-CAM + + was utilized for generating these heatmaps (Fig. 3: Grad-CAM + + heatmaps illustrating explainable AI predictions for pleural motion classification).

To assess clinical interpretability of Grad-CAM + + heatmaps, five of the expert clinicians rated 48 AI-generated heatmaps. Experts indicated whether the hotspot matched clinically relevant anatomical regions and ultrasound signs, crucial for PTX diagnosis [43] (binary rating: match vs. no-match). These 48 heatmaps corresponded to the same 48 benchmark cases used for the human expert diagnostic comparison. During heatmap relevance rating, experts were blinded to the AI output class label and to the ground truth diagnosis.

### Expert panel for benchmarking

To benchmark the AI against human performance, we assembled an expert panel of 11 physicians specializing in point-of-care ultrasound (POCUS). These experts were drawn from five major teaching hospitals and each has over ten years of experience in LUS (notably exceeding typical experience levels in prior studies, which often required > two years) [44]. Many serve as instructors in national ultrasound training programs, indicating a high level of expertise.

For the evaluation, a separate set of 48 LUS cases (B-mode video clips with their corresponding generated M-mode images) was prepared. These cases were not used in AI model training or validation, ensuring an unbiased comparison. To clearly define binary diagnostic decisions for the expert panel, cases demonstrating normal lung sliding (seashore sign), subtle lung sliding, or lung pulse (T-lines sign) were classified as ‘No PTX.’ Conversely, cases displaying absence of lung sliding (barcode sign) or clear identification of a pneumothorax border (lung point) were classified as ‘PTX.’ The 48-case evaluation set was intentionally balanced, comprising exactly 24 PTX-positive and 24 PTX-negative cases. The 48-case benchmark set included both cadaveric and in vivo acquisitions: among the 24 PTX-positive cases, 12 originated from cadaveric models and 12 from two CT-confirmed PTX patients; the 24 No-PTX cases were all in vivo and comprised ICU patients with lung sliding and healthy volunteers with lung pulse (breath-holding). In contrast, for AI model training, we explicitly included all four distinct ultrasound categories—normal lung sliding, lung pulse, absence of lung sliding, and lung point—to ensure that the AI could robustly handle even the most challenging and subtle cases. Although the AI model provided detailed, multi-class diagnostic outputs, we simplified its results into binary (PTX or No PTX) classifications for direct comparison to the binary human expert decisions. The expert panel independently reviewed these cases under a controlled protocol: all identifying patient information was removed and the experts were blinded to the correct diagnosis and to the overall prevalence of PTX in the set. Each expert was provided with both the B-mode video and the derived M-mode image for each case. They were instructed to evaluate each case and make a binary determination (PTX present or absent). Detailed instructions were given, including standardized criteria for diagnosis and guidance on viewing conditions (e.g. minimum monitor requirements), to ensure consistency. Experts recorded their findings in a structured format, noting their diagnosis for each case and answering a few questions such as which mode (B-mode vs. M-mode or both) they relied on more for that case and their confidence level. Responses were collected in individualized encrypted spreadsheets to maintain blinding between experts. After all experts completed the review, their decisions were aggregated for analysis. We compared the AI model’s predictions on the same 48 cases to the experts’ decisions.

#### Expert-set construction and workload considerations

To benchmark clinicians under a feasible reading workload while avoiding reader fatigue, we assembled a balanced 48-case evaluation set (24 PTX, 24 No-PTX). Cases were randomly sampled from subjects not used in training or validation, with stratification by ultrasound sign (barcode, lung point, seashore, lung pulse) to include both straightforward and intentionally challenging scenarios (e.g., breath-hold lung-pulse). Each expert reviewed 48 B-mode clips and the corresponding 48 M-mode images (96 media total) and completed a brief per-case questionnaire on mode reliance and confidence.

## Statistical analysis

Sensitivity and specificity was defined as the proportion of true positives among those classified as positive and the proportion of true negatives among those classified as negative, respectively. Confidence interval for these values were calculated using the exact Clopper-Pearson method.

To carry out comparisons, mixed-effects logistic regression was used, modelling the positive outcome with the subset of data that is indeed positive to investigate sensitivity, and modeling negative outcome with the subset of data that is indeed negative to investigate specificity. The advantage of this approach, described by Coughlin et al. [45], is that it allows the investigation of the impact of different covariates on sensitivity and specificity, with the additional possibility to use arbitrary functional forms (including interactions, for instance). To evaluate diagnostic performance, the fixed effect in the regression model was an indicator of the evaluator type (human expert vs. AI model). In extended analyses, evaluator type was further categorized as human expert using B-mode, human expert using M-mode, or AI model. To assess performance by ultrasound mode, the fixed effects included the evaluator (human evaluator ID or AI model) and the ultrasound mode used (B-mode or M-mode). To investigate performance differences between cadaveric and live patient scans, the fixed effects again included evaluator type (Human-B, Human-M, or AI) and a scan-type indicator (cadaveric vs. live). Finally, to evaluate expert mode preferences, the fixed effect was an indicator of whether the ultrasound mode used corresponded to the evaluator’s preferred mode. For total clarity the used codes in R are available here:

To account for correlation of evaluations within subjects, test cases and imaging modes, a random intercept was added to these three factors, except for cases when they appeared among the explanatory variables as fixed effects. To assess significances, likelihood ratio χ^2^ tests were used.

We used Fleiss’ kappa for inter-rater reliability (B-mode and M-mode diagnostics and Grad-CAM + + heatmaps).

All tests were two-tailed with an alpha level of 0·05, and exact p-values were explicitly reported throughout the manuscript.

Calculations were carried out under the R statistical program package version 4·5·0 using packages epiR version 2·0·67 and lme4 version 1·1–37.

## Results

### Diagnostic performance: AI model vs. human experts

Using the balanced test set (24 pneumothorax cases, 24 non-pneumothorax cases), the AI ensemble achieved perfect diagnostic performance, with a sensitivity of 100·0% (95% CI: 85·8%—100·0%) and specificity of 100·0% (95% CI: 85·8%—100·0%). In contrast, individual human experts, while highly sensitive on average, showed lower specificity. Table 1 summarizes the basic performance metrics for the AI model and the human experts (disaggregated by ultrasound mode) as computed from the dataset:

**Table 1:** Summary of basic performance metrics of the AI ensemble vs. human experts. Human results are shown separately for B-mode and M-mode interpretations. For human experts, confidence interval pertains to the confidence interval for the average performance of all experts combined

Metric	AI Model, M-mode (95% CI)	Human Experts, B- mode average (95% CI) [range]	Human Experts, M- mode average (95% CI) [range]
Sensitivity	100·0% (85·8%–100·0%)	95·8% (92·7%–97·9%) [83·3%–100·0%]	92·8% (89·0%–95·6%) [79·2%–100·0%]
Specificity	100·0% (85·8%–100·0%)	81·0% (75·8%–85·6%) [54·2%–100·0%]	73·5% (67·7%–78·7%) [45·8%–100·0%]
Accuracy	100·0% (92·6%–100·0%)	88·4% (85·4%–91·0%) [75·0%–93·8%]	83·1% (79·7%–86·2%) [64·6%–93·8%]

In contrast to the AI’s perfect diagnostic performance, human experts, while at least one managed to achieve perfect score with each mode and metric, exhibited worse performance on average, especially for specificity.

These results confirm that the AI outperformed the experts, primarily by reducing false positives. AI and human experts were statistically compared in three different approaches.

The first model aggregated the overall human performance using only measurements in M-mode and compared it with the AI model; sensitivity showed no significant difference (*p* = 0·09516), while specificity demonstrated a significant advantage in favor of the AI model (*p* = 0·02466).

In the second model, the human performance was aggregated across both ultrasound modes (M-mode and B-mode combined) and again compared to the AI; similarly, sensitivity differences were non-significant (*p* = 0·1338), whereas specificity significantly favored the AI (*p* = 0·0194).

**Fig. 4:**
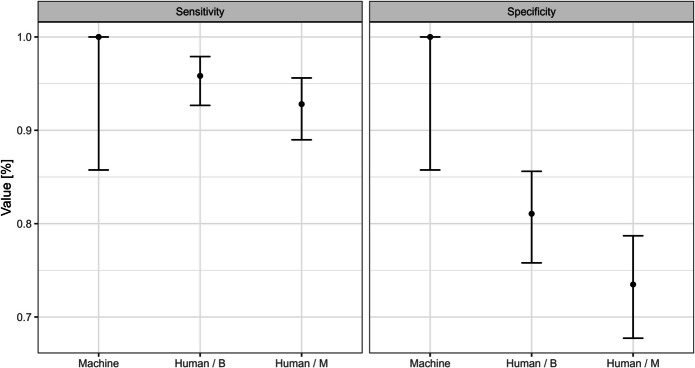
Comparative diagnostic performance of human experts and AI model. The Figure shows the sensitivity and specificity of AI model and humans (aggregated) with different modes. Error bars represent 95% confidence intervals

The third, more detailed model assessed AI versus human with B-mode versus human with M-mode performance; for sensitivity the differences in these three approaches were non-significant (*p* = 0·09112), but for specificity, the differences were significant (*p* = 0·001301) (Fig. 4: Comparative diagnostic performance of human experts and AI model.)

**Fig. 5:**
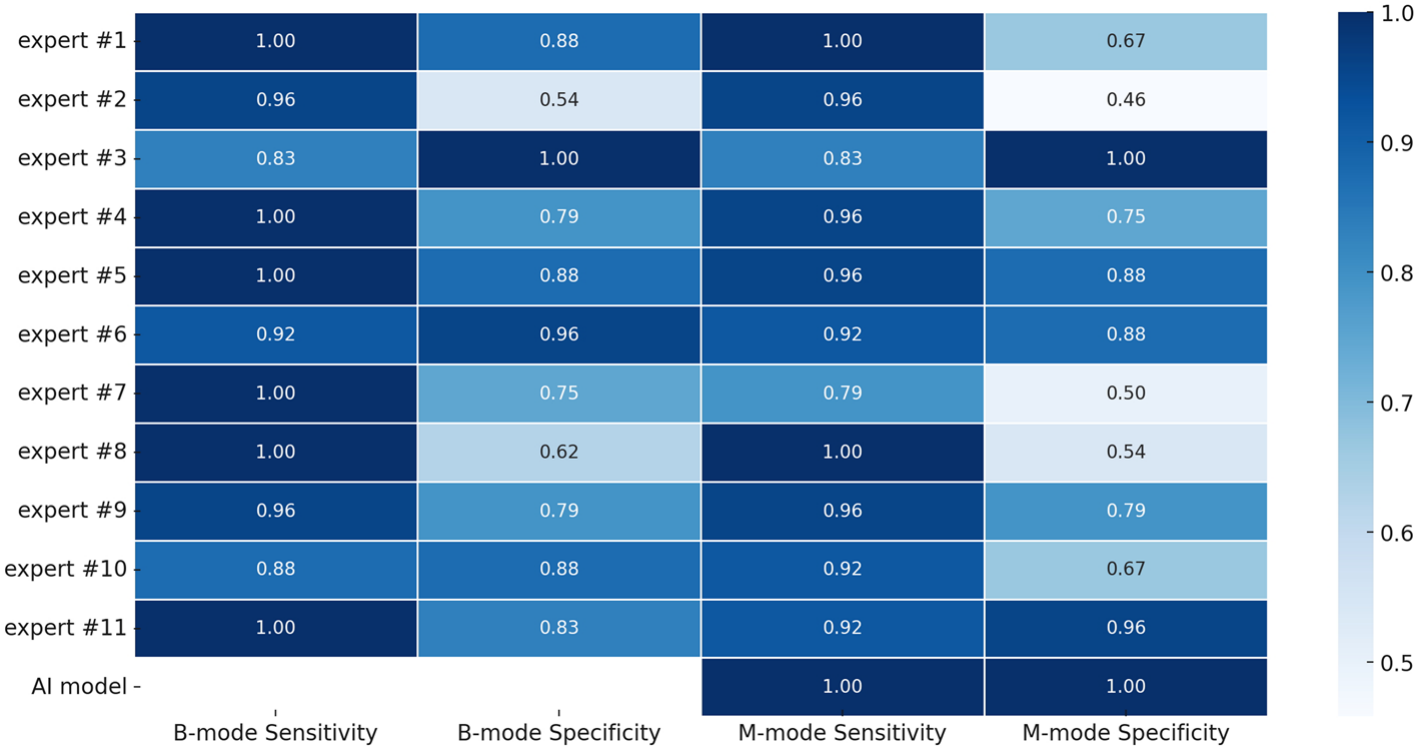
Overall sensitivity and specificity performance of individual experts and AI model (Heatmap). The heatmap depicts the individual diagnostic performance (sensitivity and specificity) of the 11 expert clinicians compared with the AI model, separately assessed for B-mode and M-mode lung ultrasound. Each cell's intensity corresponds to performance, with darker blue indicating higher performance. The AI model's performance, shown in the bottom row, demonstrates consistently high diagnostic accuracy across both imaging modes. This visualization underscores variability among human experts, especially in specificity, and highlights the AI's superior and stable performance across conditions

No human expert achieved the AI’s combination of high sensitivity *and* high specificity on this dataset. In fact, no expert found a pneumothorax that the AI missed, indicating the AI caught all true positives that any expert could detect. Even the best human experts had to trade off sensitivity and specificity: six clinicians reached 100·0% sensitivity but among them, even the best specificity was 87·5%, while a single expert achieved 100·0% specificity, but only with 83·3% sensitivity. The AI, by contrast, operated at the ideal point (100·0%, 100·0%), essentially matching the consensus of an 11-expert panel on each case. (For context, a majority vote of all 11 experts per case would have yielded performance comparable to the AI, whereas no single expert matched it.) These results underscore that the AI model achieved a level of accuracy not attained by any individual human, with significantly fewer false alarms while detecting at least as many true pneumothoraces. (Fig. 5: Overall sensitivity and specificity performance of individual experts and AI model.)

### Performance by ultrasound mode (B-mode vs. M-mode)

Human experts’ diagnostic performance differed by imaging mode. B-mode ultrasound (the conventional 2D mode) enabled higher accuracy than M-mode for the clinicians (Table 1).

With respect to sensitivity, there was no significant interaction between mode and expert (*p* = 0·2619), and the difference in modes was not significant (*p* = 0·09105). For specificity, there was no significant interaction between mode and expert either (*p* = 0·1489), but the difference between B- and M-modes was significant (*p* = 0·00337). That is, experts were more likely to misidentify pneumothorax on M-mode, primarily due to more false-positive calls. Most experts showed equal or better performance with B-mode, indicating a consistent mode-effect.

By contrast, the AI model was evaluated only on M-mode images, yet, it still excelled. Notably, the AI’s M-mode sensitivity (100·0%) was on par with the experts’ B-mode sensitivity, and the AI’s specificity far exceeded even the B-mode expert specificity.

In other words, even though M-mode was generally the less favored modality for humans, the AI proved capable of interpreting M-mode with expert-level sensitivity and superior specificity. These findings confirm that while human clinicians perform best with B-mode images (maybe benefitting from the direct visual lung sliding cues), the AI can leverage M-mode information extremely effectively, reaching performance that surpasses humans in either mode.

### Performance on cadaveric vs. live patient scans

We next verified whether the source of the ultrasound (cadaveric model vs. live patient) affected diagnostic performance. Since all cadavers were positive, only sensitivity could be evaluated among them. Table 2 summarizes the sensitivity outcomes by specimen type and mode:

**Table 2:** Sensitivity for pneumothorax detection, stratified by imaging source (cadaveric models vs. living patients) and ultrasound mode. For human experts, confidence interval pertains to the confidence interval for the average performance of all experts combined with the given mode

Specimen Type	AI model (95% CI)	Human experts, B-mode, average (95% CI)	Human experts, M-mode, average (95% CI)
Cadaveric scans	100·0% (73·5%–100·0%)	97·8% (93·5%–99·5%)	91·7% (85·6%–95·8%)
Live patient scans	100·0% (73·5%–100·0%)	93·9% (88·4%–97·3%)	93·9% (88·4%–97·3%)

We found that cadaveric ultrasounds yielded slightly different trends than live patient ultrasounds. On cadaveric scans, B-mode sensitivity was extremely high (97·7%) while M-mode sensitivity was a bit lower (91·7%). For live patient scans, sensitivities were equal between B and M (94·0%) modes. Statistically, there was no significant interaction between specimen type and mode (*p* = 0·213) – in other words, the difference among cadaveric and live scans did not significantly differ according to mode. This difference itself was also not significant in a model without interaction (*p* = 0·9865). These results suggest that the AI and experts performed consistently on cadaveric data versus real patient data, supporting the validity of using cadaver models for training/evaluation. Any minor differences (such as the slightly higher B-mode sensitivity on cadavers) were not statistically significant and likely reflect the small sample or subtle ease of detecting an induced pneumothorax under ideal conditions.

### Expert mode preferences and diagnostic consistency

The dataset also recorded each expert’s mode preference for each case (i.e. which mode—B-mode, M-mode or both—they relied on more for that case) and whether using their preferred mode improved their performance. Across all 1056 interpretations (48 cases ×11 experts ×2 modes), experts indicated no particular mode preference (either judging both or none as useful) in 57·0% of cases, preferring B-mode in about 23·1% and M-mode in 19·9% (these percentages correspond to 602, 244, and 210 out of 1056 interpretations, respectively).

When an expert did have a preferred mode, one might expect they’d perform better with that mode. Indeed, we found a slight increase in accuracy when experts used their preferred mode, but this was not significant (OR = 1·18, *p* = 0·772 for sensitivity; OR = 1·26*, p* = 0·535 for specificity).

Overall accuracy did not markedly differ by preference category: those who preferred B-mode had an average sensitivity of 91·5% (95% CI: 85·0%–95·9%) versus 97·2% (95% CI: 92·0%–99·4%) for those who preferred M-mode; for average specificity these were 77·0% (95% CI: 68·6%–84·0%) and 71·2% (95% CI: 61·4%–79·6%) respectively. The difference was significant for sensitivity (*p* = 0·0275), but not for specificity (*p* = 0·5717).

In other words, having a particular favored mode did not appreciably improve diagnostic success in aggregate. Both B-mode and M-mode, when used, could be „utilized to high effect” by the experts.

This finding suggests that while individual experts varied in which mode they felt more comfortable with, they were generally capable of diagnosing pneumothorax with either modality.

Notably, in ~29% of cases where an expert expressed a preference, the diagnosis was actually wrong in the preferred mode. This underlines that a clinician’s intuition about which mode is best isn’t infallible – sometimes the alternative mode would have caught the finding. Overall, the consistency analysis indicates that expert performance was robust across modes, and an absence of strong preference was not detrimental. For the AI, which applies a uniform algorithm to M-mode images, such preference bias is a non-issue, and it provides consistent results without mode-dependent variability.

It is worth noting, however, that despite the minimal impact of mode preference on overall accuracy, there was a statistically significant reduction in specificity when experts used M-mode compared to B-mode. This finding, discussed in detail in the previous section, highlights that while experts might feel comfortable with either mode, objective performance metrics did consistently favor B-mode, especially regarding specificity.

### Inter-observer variability and case difficulty

There was considerable variability both in individual expert performance and in the difficulty of each case.

Individual experts varied in their success rates: sensitivities ranged roughly from 79·2% up to 100·0%, and specificities from 45·8% up to 100·0% among the 11 clinicians. Most experts clustered near the high-sensitivity end – six of 11 had perfect sensitivity with at least one mode, and two among them achieved it with both modes. However, this often came at the expense of specificity. For instance, one expert with 100% sensitivity had only 54% specificity (falsely labeling ten plus normal cases as PTX). Conversely, there was a single expert with perfect specificity (with both modes), but this expert missed four pneumothoraces (83·0% sensitivity). This trade-off pattern – some experts being more “vigilant” (high sensitivity, low specificity) and others more “cautious” (high specificity, lower sensitivity) – is evident and is typical in diagnostic tasks. The average individual accuracy of experts ranged from 64·6% up to 93·8%, as noted above.

Inter-rater reliability among the expert panel, assessed by Fleiss' kappa, was substantial for B-mode imaging (κ = 0·695) and moderate for M-mode imaging (κ = 0·577).

Case difficulty also varied widely. Some LUS cases were straightforward – in fact, 18 out of 48 cases (37·5%) were correctly diagnosed by all 11 experts (100·0% consensus) in both B-mode and M-mode. In several additional cases, all but one expert got the diagnosis correct (≈90% agreement). These images with clear findings had virtually no interobserver disagreement. By contrast, a few challenging cases proved confusing even to seasoned experts.

We identified the five most challenging images based on expert diagnostic accuracy and specificity. Upon decrypting the image IDs to investigate their original sources and characteristics, all five images were identified as originating from cases described as "breath-holding," explicitly mimicking pneumothorax (PTX). These images were intentionally included in our study due to the recognized clinical difficulty associated with subtle pleural motions on B-mode imaging and their representation as the "T-line sign" in M-mode imaging. Such subtle pleural movements are notoriously challenging for clinicians to detect accurately, reinforcing the critical need for focused training. This finding underscores that these cases possessed ultrasound characteristics particularly prone to inducing false-positive errors, challenging the diagnostic skills of even experienced clinicians.

These findings strongly support our baseline hypothesis that targeted training of the AI model for subtle pleural motions and T-line signs is justified and beneficial. Notably, our AI model was not misled by these subtle motions, confirming the validity and effectiveness of our targeted training approach.

### GRAD-CAM + + heatmap for explainability

To provide transparency and clinical interpretability to our ensemble AI model, we applied the Grad-CAM + + method to visualize the image regions most influential in the AI's decision-making process. We specifically aimed to determine whether these visual explanations were clinically meaningful, i.e., whether the AI's highlighted areas corresponded closely to the anatomical regions and ultrasound M-mode signs that clinicians would focus on when diagnosing pneumothorax using ultrasound.

Five of our experts assessed the clinical relevance of the AI-generated Grad-CAM + + ensemble heatmaps for all cases in the test set. Experts rated the highlighted regions explicitly, indicating whether the AI hotspots matched clinically relevant anatomical landmarks and ultrasound signs, essential for accurate pneumothorax diagnosis. The inter-rater reliability among these experts was substantial, with a Fleiss' Kappa of 0·750, underscoring strong consensus regarding the clinical validity of the AI's visual explanations.

Overall, experts rated 84·2% (95% CI: 79·0%–88·2%) of the Grad-CAM + + hotspots as clinically relevant. This high proportion demonstrates that the AI model's decision-making process aligns effectively with clinical expectations and highlights the robustness of our explainable AI approach. The results suggest that Grad-CAM + + not only adds transparency but also meaningful interpretability, enhancing trust in the model's clinical utility [41, 46].

## Discussion

Given the modest benchmark size (n = 48), these perfect point estimates should be interpreted cautiously; the corresponding exact 95% confidence intervals remain relatively wide (e.g., 85·8%–100·0%), and larger prospective multicenter validation studies are warranted.

In this study, we rigorously verified that a soft-voting AI ensemble can match or exceed expert clinician performance for pneumothorax detection on lung ultrasound. The key finding is that the AI achieved perfect sensitivity and specificity (100·0%), effectively combining the strengths of human observers without their trade-offs. Eleven experienced clinicians, drawn from five hospitals, served as a robust benchmark – they detected ~95% of pneumothoraces overall, but individual performance varied, especially in specificity. Some experts prioritized sensitivity (avoiding misses at the cost of false alarms) while others were more conservative, leading to false negatives.

The AI, however, demonstrated high sensitivity and high specificity concurrently, operating as if it were an “idealized expert” who catches nearly all true pneumothoraces while seldom crying wolf on normals. This consistency resulted in significantly fewer false positives than any single human – a clinically crucial advantage, since unnecessary interventions (e.g. chest tube for a false-positive PTX) can be harmful. At the same time, the AI did not miss pneumothoraces that experts could find, ensuring no compromise in safety (no increase in false negatives).

This suggests that the AI could reduce false alerts and the downstream confirmatory tests or treatments they provoke.

Another notable result is the lack of statistically significant difference in sensitivity between the AI and experts – in other words, the best human doctors were just about as sensitive as the AI, and the AI did not significantly “miss” more pneumothoraces than humans. This finding reinforces that our expert panel was a strong comparator (several had perfect sensitivity), and it took an AI of this caliber to perform on par in terms of picking up every pneumothorax. However, the fact that the AI maintained extremely high specificity concurrently is what set it apart (experts had to trade-off one for the other).

Statistically, the AI’s specificity advantage over the experts was significant. Even those experts with relatively few false positives still trended worse than the AI in specificity. In clinical terms, this means the AI can provide confidence in ruling out pneumothorax when it says “No PTX,” without incurring the penalty of many false alarms – a balance that even seasoned clinicians struggle to achieve. This consistency and reliability of the AI system is a major clinical asset: it behaves like an ensemble of multiple opinions, effectively providing an automated consensus. Indeed, as shown in the results, the model’s performance was comparable to a hypothetical majority vote of 11 experts on each case. Such consensus-level accuracy, delivered instantly by the AI, could be transformative in acute settings where getting an eleventh (or even a second) human opinion in real-time is impractical.

Expert variability was clearly evident in our analysis, underscoring why an AI assistant could be valuable. While all experts were experienced, their individual accuracies ranged from ~75% to ~94%. Each brought their own decision threshold and interpretative style, leading to variability in outcomes (some missed findings that others caught, and vice versa). This inter-observer variation can impact patient care, as the diagnosis might depend on which clinician reads the ultrasound [44]. Our data showed, for example, one expert false-alarmed on nearly half of normals, while another missed 4 of 24 pneumothoraces. The AI’s advantage is that it applies a uniform, optimized criterion to every case, eliminating this variability. In effect, it standardizes the diagnostic performance to a very high level, which could reduce the luck-of-the-draw in which physician is on call. Moreover, the AI was error-free on many of the easy cases and remained competent on the hardest cases, whereas humans showed more divergence on those challenging images.

Notably, there were images in which a large fraction of experts were incorrect (nearly half misdiagnosing in some instances), and these are scenarios where an AI opinion could flag the disagreement or prompt a second look. The AI did not fall for some of the pitfalls that tricked multiple experts (e.g. subtle “T-line signs” in M-mode that mimicked a “barcode sign”), indicating a robustness to certain false- positive traps. This kind of AI consistency – performing uniformly across cases of varying difficulty – is a significant benefit for maintaining diagnostic quality.

It is also supported by the model’s use of objective features; for instance, our AI utilized Grad-CAM + + visual explanations that consistently highlighted the real “diagnostic hot spot” regions on M-mode images when making decisions, with a strong consensus on experts’ opinion. Such consistency in what the model “looks at” likely contributes to its consistent outputs and builds trust that the AI is focusing on anatomically relevant cues, not spurious patterns.

The study also provided insight into B-mode vs. M-mode usage. As expected, most clinicians found B-mode slightly more intuitive, and their performance was better with B-mode images (higher specificity and confidence). Some experts explicitly preferred B-mode in many cases, citing the direct visualization of lung sliding and pleural interface. M-mode strips, while valuable for depicting motion as a graph, can be abstract and were associated with more false positives (e.g. misreading motionless artifacts as a “barcode” of PTX). Interestingly, however, about 20% of cases saw experts preferring M-mode, often when B-mode was equivocal – these experts leveraged M-mode to confirm subtle absent motion. Our analysis found no substantial accuracy penalty for using one’s favored mode to the other, meaning a skilled interpreter could use M-mode nearly as effectively as B-mode, which is in line with previous studies [11]. Despite the minimal impact of mode preference on overall accuracy, there was a statistically significant reduction in specificity when experts used M-mode compared to B-mode.

The AI’s performance being based solely on M-mode underscores this point: the information content in an M-mode image is sufficient for excellent diagnosis, but humans may require training to interpret it as reliably [15]. In fact, the AI’s success suggests that certain subtle signatures in M-mode are highly indicative, and the model exploited these consistently.

The analysis explicitly demonstrates that images involving complex breath-holding scenarios and subtle pleural motions significantly reduce human diagnostic accuracy. This underscores the necessity for targeted AI training and enhanced clinical education to reliably manage these challenging diagnostic scenarios.

From a clinical standpoint, this means an AI could augment M-mode interpretation, serving as a tireless second reader for what many consider a “backup” modality. It also hints that incorporating AI into the ultrasound workflow could give clinicians more confidence to use M-mode when appropriate, knowing an expert-level interpretation is available. Furthermore, the lack of strong preference impact on outcomes implies that both modes have merit – an ideal system might toggle between or combine B-mode and M-mode analysis. Indeed, some of our experts without a strong bias performed just as well, indicating that proficiency in both modalities is achievable. The AI currently uses M-mode due to training design, but a future extension to integrate B-mode frames could potentially yield even greater accuracy or usability (e.g. analyzing a B-mode clip and its derived M-mode in parallel).

Clinically, the verified findings are encouraging. An AI system with 100·0% accuracy for pneumothorax on ultrasound could rival the diagnostic gold standard (CT scans) in accuracy, yet provide results instantly at the bedside. This has direct implications for critical care and emergency medicine [2]: faster and more reliable PTX diagnosis enables prompt treatment (needle decompression or chest drain) and avoids delays or unnecessary radiation from confirmatory CT or X-ray. The reduction in false positives is particularly relevant – pneumothorax rulings often err on the side of caution, but an AI that markedly lowers false alarms could prevent unnecessary invasive procedures (e.g., not every absent lung sliding will be overcalled as PTX if the AI identifies alternative signs of a normal lung). Likewise, maintaining near-perfect sensitivity means we can trust the AI not to overlook a real pneumothorax, which is paramount for patient safety. The consensus-level performance of the AI also provides a safety net: it’s as if you have a panel of experts consulting on each scan, which could be valuable in settings with less experienced operators. This highlights that the AI can serve as a force multiplier – even in centers with strong ultrasound expertise, it can push performance to an even higher level, and in environments with less expertise, it could be even more transformative.

There were no indications that the AI’s performance was limited to a particular sub-group of images – it did well on both cadaveric simulation scans and real ICU patient scans as well as healthy volunteers scans. Together, these points illustrate that the AI provides consistent, reliable detection of pneumothorax and could standardize care by mitigating human variability.

## Study limitations

This study has certain limitations. First, although the dataset is among the largest human lung ultrasound collections for pneumothorax detection, it originated from a single-center, potentially affecting broader generalizability. Multicenter validation remains essential to confirm the robustness of our findings across diverse patient populations and clinical environments. Second, despite employing real-world clinical data and precisely generated M-mode images identical to standard ultrasound machine outputs, the retrospective nature of both AI model validation and expert assessments differs inherently from prospective bedside decision-making scenarios. Although we have developed a clinically usable real-time bedside version of our model (see Supplementary Material video), its impact on clinical outcomes and integration into routine workflows necessitates prospective evaluation. Additionally, while the AI model's current use of M-mode images demonstrates strong diagnostic performance, incorporating B-mode analysis might further enhance overall diagnostic capabilities and clinical usability. Finally, our benchmarking involved a panel of 11 highly experienced clinicians, significantly exceeding typical expert numbers in similar studies, yet still represents subjective diagnostic variability inherent to human interpretation. Future studies involving larger expert groups and varied operator experiences would further solidify these findings.

## Conclusions

We conclude that the AI ensemble model offers diagnostic performance on par with, or superior to, experienced human clinicians for pneumothorax detection in lung ultrasound. The results show the AI achieving 100·0% sensitivity and 100·0% specificity on a challenging, balanced test set – an accuracy level no single expert could reach. Human experts were highly sensitive (~95% on average) but showed variable specificity and inter-observer inconsistency, especially when using M-mode alone. The AI, in contrast, was consistently accurate across all cases and conditions, effectively eliminating the usual sensitivity–specificity trade-off. Statistically, the AI’s performance was significantly better in terms of specificity (meaning fewer false positives) than the experts, while maintaining equivalent sensitivity.

This robust performance was observed for both cadaveric model scans and real patient scans, indicating strong generalizability within the tested domain. In practical terms, an AI-driven interpretation of lung ultrasound could ensure that pneumothorax is detected with extremely high reliability, matching CT-level confidence but at the bedside and without radiation [8, 47]. Such a tool can elevate point-of-care ultrasound from an operator-dependent skill to a more standardized diagnostic modality supported by AI.

From a clinical perspective, these findings highlight a potential paradigm shift: an AI system can function as a perpetual “expert colleague” providing a second opinion on ultrasound images, reducing diagnostic errors. For pneumothorax management, this means more consistent early detection and fewer unnecessary procedures from false alarms. The expert panel comparison demonstrated that even well-trained humans vary in their interpretations – the AI can help bridge this variability, offering a safety net especially in resource-limited or high-stress settings.

Our findings clearly validate the diagnostic difficulty posed by subtle pleural-motion variants associated with breath-holding, explicitly confirming the necessity of targeted AI training on these challenging scenarios, as initially hypothesized. Additionally, these results underline the importance of increased clinical emphasis on recognizing such subtle signs to enhance clinicians' diagnostic accuracy.

It is noteworthy that the AI achieved its performance using only M-mode images, a testament to the information richness of that format and the model’s capability; meanwhile, human experts may have benefitted from B-mode context as well. This suggests future systems that incorporate both modalities may further improve performance or user confidence.

The demonstrated clinical relevance and interpretability of the Grad-CAM + + visual explanations underscore their importance in fostering clinician trust, facilitating acceptance, and enhancing practical integration of our AI solution into real-world diagnostic workflows [46].

## Supplementary Information

Supplementary Material 1.

## Data Availability

The datasets generated and/or analyzed during the current study are not publicly available due to institutional policies and according to the ethics approval but are available from the corresponding author ([orosz.gabor@semmelweis.hu](mailto:orosz.gabor@semmelweis.hu)) on reasonable request. The underlying code for this study (and all training/validation datasets, weigths and biases) is not publicly available but may be made available to qualified researchers on reasonable request from the corresponding author ([orosz.gabor@semmelweis.hu](mailto:orosz.gabor@semmelweis.hu)). The complete statistical analysis with code in R is available to qualified researchers on reasonable request from the corresponding author ([orosz.gabor@semmelweis.hu](mailto:orosz.gabor@semmelweis.hu)) (https://github.com/ferenci-tamas/lung-ultrasound-pneumothorax-detection).

## Abbreviations

ICU: Intensive Care Unit
Grad-CAM + +: Gradient-weighted Class Activation Mapping
PTX: Pneumothorax
LUS: Lung Ultrasonography
POCUS: Point-of-Care Ultrasonography
M-mode: Motion mode ultrasound
B-mode: Brightness mode ultrasound
